# Double-Recycled Reclaimed Asphalt Pavement: A Laboratory Investigation at Low Temperatures Based on Different Mathematical Approaches

**DOI:** 10.3390/ma13133032

**Published:** 2020-07-07

**Authors:** Ki Hoon Moon, Augusto Cannone Falchetto

**Affiliations:** 1 Pavement Research Division, Korea Expressway Corporation, Hwasung 18489, Korea or zetamkh@ex.co.kr; 2 Department of Civil and Environmental Engineering, University of Alaska Fairbanks, Fairbanks, AK 99775, USA

**Keywords:** SRRAP, DRRAP, low-temperature performance, BBR mixture test, re-recycling

## Abstract

Using recyclable materials in asphalt pavement is a fundamental design approach not only for limiting the environmental impact of the construction industry, but also for reducing the overall costs of the road infrastructures. Over the past years, road agencies have developed different policies to incorporate various types of recyclable material into conventional asphalt mixtures. reclaimed asphalt pavement (RAP) is one of the most highly recycled construction materials. However, the aged RAP binder and its stiffer and brittle characteristics compared to the fresh binder may negatively affect the performance of the recycled mixture, especially when operating in cold climates. In this study, the low-temperature response of asphalt mixture prepared with single-recycled RAP (SRRAP) and double-recycled RAP (DRRAP), prepared in the laboratory, is experimentally investigated based on creep testing performed with the bending beam rheometer (BBR). Then, the data were analyzed based on three simple mathematical models to extract information on material behavior. Finally, a new indicator named thermal stress factor (*TFS*) on low-temperature response is proposed. Relatively poorer performance was observed from SRRAP mixture compared to the asphalt mixture prepared with virgin material. However, the low-temperature response between SRRAP and DRRAP did not present significant differences. The values of *TFS* support the experimental results and suggest the possibility of considering re-recycling technology for further research with the objective of a possible application in the asphalt pavement industry.

## 1. Introduction

In the asphalt pavement industry, recyclable materials such as reclaimed asphalt pavement (RAP), end of life asphalt shingles, construction and demolition waste, steel slag, waste rubber and waste tire have been widely considered for pavement construction over the years [1,2,3,4,5,6,7,8,9,10,11,12,13]. Among these materials, RAP has found remarkably increased applications in the past decades [6,7,14,15].

RAP is obtained from the milling process on the existing asphalt pavement and generally consists of particles made of aggregate and aged asphalt binder [1,2,3,4,5,6,7,9,14,15]. Two main advantages can be identified from the use of RAP: first, approximately 0.8–1.1% of the asphalt binder amount can be saved during the mixture production when incorporated with virgin material [1,2,3,4,5,6,7,8,16] compared to the conventional asphalt mixtures and second, due to the incorporation of RAP in the mix design, aged pavement layers can be reused mitigating the overall environmental impact of the construction process [11,12,13,14]. During 2017, approximately 76.2 million tons of RAP were successfully reused for pavement applications in the U.S. with RAP producers being the most effective in recycling up to 99% of the RAP [17]. A much large amount of recycled asphalt material was produced in China during the recent past reaching the value of 160 million tons of RAP annually [18]. In the case of Europe and South Korea, approximately 47 and 21 million tons of RAP are annually produced and recycled for asphalt pavement construction, respectively [18,19,20]. In South Korea, especially, several large-scale asphalt overlay projects are planned; this is because an increasing portion of the road network that was built during the 1980s and 1990s is experiencing severe aging conditions (this includes not only asphalt, but also concrete pavement) [21,22]. Therefore, due to the current construction trend, pavement management agencies in South Korea and other countries are facing the following two issues: first, an increase in the construction costs and second, a larger demand for incorporating a higher amount of RAP in pavement projects [23,24,25,26,27]. Given these circumstances, considering not only a single recycling approach––single recycled RAP (SRRAP)––but also the possibility of adopting re-recycled RAP––double-recycled RAP (DRRAP)––was explored in some countries such as the United Kingdom and Japan [23,24,25,26,27].

It is well known that RAP mixtures tend to present higher stiffness and potentially increased brittleness compared to conventional hot mix asphalt (HMA) mixtures. This may imply relatively better performance against rutting; however, potentially poorer resistance to fatigue and thermal cracking may be expected [1,2,3,4,5,6,7,9,10,14,16]. Over the years, several studies were performed both in the laboratory environment and in the field to investigate the use of RAP. Based on these efforts, the use of 15–25% of SRRAP was recommended when producing recycled mixture [6,7,9,12,14,16,23,24,25,26,27]. Yoshinake et al. [23] conducted a field investigation over a time span of 15 years to evaluate the effect of DRRAP on actual pavement performance. As a result, satisfactory performance was observed when using DRRAP in comparison to SRRAP and conventional hot mix asphalt (HMA) mixture. Su et al. [24] investigated rutting, abrasion and fatigue behavior of conventional asphalt mixture and DRRAP with the latter presenting a comparable response to the virgin mixture. Heneash and co-workers comprehensively studied the fatigue performance of DRRAP [25]. A deterioration in fatigue response was experienced during the first recycling process; however, no further reduction in fatigue performance was found after the second round of recycling suggesting the possibility of considering the application DRRAP technology in actual pavement construction projects. Hunger and Kawakami [27] experimentally addressed various properties of DRRAP mixtures, including moisture sensitivity, resistance to deformation and fatigue behavior. They concluded that up to 40% of recycled material can be incorporated in the design of double-recycled mixtures to be used in the pavement without any significant performance deterioration compared to the conventional HMA. Recently, the combination of regular asphalt binders with double coated recycled concrete aggregates (DCRCAs) was evaluated by Kareem et al. [28,29] through various performance tests such as rutting, fatigue and dynamic performance evaluation. Higher resistance against rutting and relatively lower performance at low temperatures were observed which suggests that double-coated and recycled aggregate from concrete can be potentially used in asphalt mixture production in the future.

Even though the use of DRRAP was considered and research on the topic was performed, most of these studies were based on simple experimental works with limited field evaluation. It is known that poorer performance at low temperatures may be expected compared to the conventional asphalt mixtures when incorporating DRRAP (or SRRAP) in the design formula [6,7,12,14,16]. However, not many studies consistently addressed the effect of DRRAP on the low-temperature performance both experimentally and through the application of different mathematical approaches for the analysis of the data.

## 2. Objective and Research Approach

**Figure 1:**
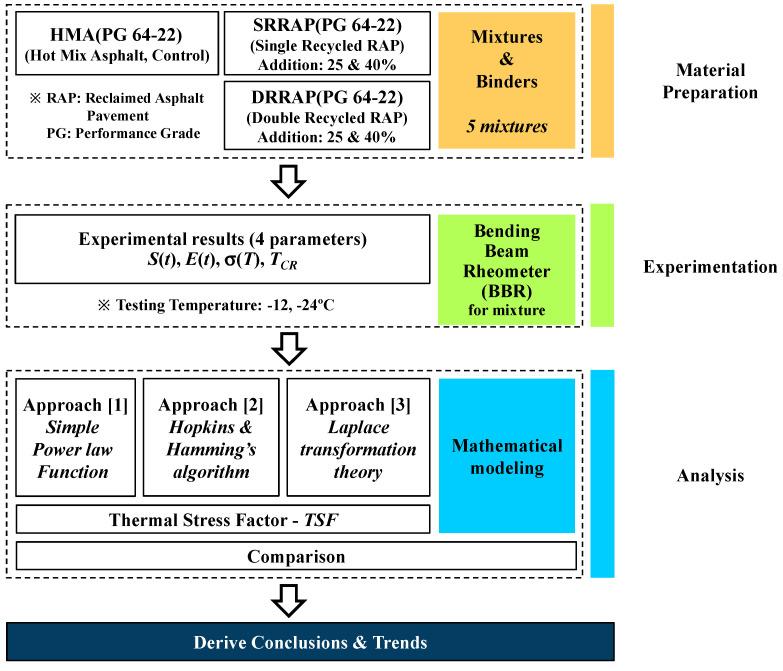
Schematic flow chart of the research approach.

The present work aims to evaluate the low-temperature behavior of asphalt mixture designed with a double recycling process. For this purpose, virgin, SRRAP and DRRAP mixtures were prepared in the laboratory. Then, low-temperature creep tests were performed with a bending beam rheometer (BBR) [6,30,31,32,33] and the data analyzed with three different mathematical models. Parameters such as creep stiffness, *S*(*t*), relaxation modulus, *E*(*t*), thermal stress *σ*(*T*) and critical cracking temperature, *T_CR_*, were generated for each tested asphalt mixture and compared. In addition, a simple low-temperature indicator is introduced under the name of thermal stress factor (*TSF*) to provide a simple and easy comparison tool for further analysis. Finally, the possibility of using DRRAP mixtures in actual asphalt pavement construction projects is discussed. The schematic research approach adopted in the present work is shown in Figure 1.

## 3. Material Preparation

In this study, a total of five different asphalt mixtures were prepared with an asphalt binder commonly used in South Korea presenting a performance grade PG 64–22 [34] (see Table 1). A conventional hot mix asphalt (HMA) mixture, widely adopted for surface layer in South Korea and the U.S., and presenting a Nominal Maximum Aggregate Size (NMAS) of 12.5 mm was set as reference material (i.e., Mixture A, WC: Wearing Course type-1 mixture) [35]. Mixture A was next used to produce the first and the second generation of reclaimed asphalt pavement (RAP) and corresponding mixtures with similar aggregate gradation curve compared to the control mixture (Mixture A).

**Figure 2:**
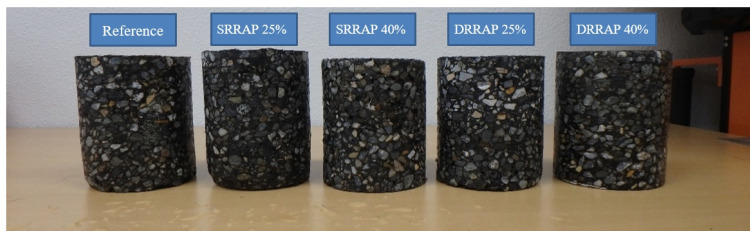
Asphalt mixture samples (two asphalt mixture samples were prepared for each mixture type).

The RAP for single and double recycling was obtained in the laboratory through artificial aging. First, the virgin asphalt mixture specimens (mixture A) were crushed in small particles close to the design NMAS and then aged by heating the mixture in the oven at 105 °C for 40 h to obtain the first generation of RAP [25]. This material (SRRAP) was next used to prepare SRRAP mixtures with SRRAP content of 25% (Mixture B) and 40% (Mixture C). These two mixtures were then crushed into lose mixture and subjected to the same aging procedure previously adopted to generate RAP material of second-generation (DRRAP) which was next incorporated in the mix design of DRRAP mixtures (mixtures D and E). To keep the experimental design within a limited number of materials, the DRRAP obtained from mixture B was used to produce mixture D with the same content of recycled material: specifically, 25% of DRRAP by mass. Similarly, the DRRAP derived from mixture C was added to mixture E at the corresponding amount of 40%. It must be remarked that the adopted aging procedure was selected as this was already applied for repeated recycling in a work from a different author [25]. Alternative aging methods can be considered as long as they are consistently used throughout the research. Nevertheless, to the knowledge of the authors, there is not unique aging protocol capable of replicate aging in the field. The latter is affected by several variables such as climate, location, weather, construction materials and techniques, among others. A summary of the mix design of the mixtures prepared for this study is presented in Table 1. The entire set of five mixtures was compacted using a Superpave gyratory compactor (SGC). Figure 2 shows the different asphalt mixtures.

## 4. Experimental Work

**Figure 3:**
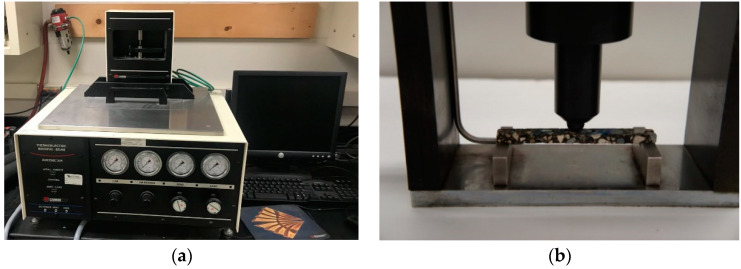
Testing setup of bending beam rheometer (BBR) mixture creep test: (**a**) BBR testing device, (**b**) mixture beam sample.

The low-temperature performance of asphalt material can provide information on the resistance to thermal cracking. For this purpose, parameters such as thermal stress [6,7,12,14,16] can be used. It is well known that thermal stress can be computed by performing low-temperature creep test with the bending beam rheometer (BBR) [36]. BBR creep test was originally developed for asphalt binder [36]; however, as demonstrated in the past, through the modification of the testing system, it is possible to perform BBR creep tests on small asphalt mixture beams [6,31]. In this procedure, the constant load *P* = 980-mN applied for 240 s (used for asphalt binder) is replaced by a higher *P* in the range 4000–6000 mN. Such a higher load is imposed on a small beam of asphalt mixture for an extended duration of 1000 s due to the higher stiffness of the material [6,30,36]. In addition, two different testing temperatures were considered to generate the master curves of the relaxation modulus, *E*(*t*), and hence the thermal stress. Figure 3 presents the testing setup, while Table 2 summarizes the testing conditions.

From the BBR measurements, two rheological parameters, creep stiffness, *S*(*t*) and *m*-value, *m*(*t*), can be calculated as [6,30,36]:

(1) $$\left\{ \begin{array}{l} S(t)=\frac{1}{D(t)}=\frac{\sigma }{\varepsilon (t)}=\frac{P\cdot l^{3}}{4\cdot b\cdot h^{3}\cdot \delta (t)}\\ m(t)=\left|\frac{d\mathrm{Log}S(t)}{d\mathrm{Log}(t)}\right|≅\left|\frac{d\left(A_{1}\cdot {\left[\mathrm{Log}(t)\right]}^{2}+A_{2}\cdot \mathrm{Log}(t)+A_{3}\right)}{d\mathrm{Log}(t)}\right| \end{array}\right.$$

where:

*S*(*t*) = time-dependent flexural creep stiffness (MPa);
*D*(t) = creep compliance (1/MPa);
*σ* = bending stress in the beam (MPa);
*ε*(*t*) = time-dependent bending strain in the beam (mm/mm);
*P* = applied constant load (mN);
*δ*(*t*) = beam deflection (mm);
*L*, *b*, *h* = beam dimensions (*L* = 102 mm, *b* = 12.7 mm, *h* = 6.25 mm);
*t* = time (s);
*A*_1_, *A*_2_, *A*_3_ = fitting constants

From Equation (1) based on linear viscoelastic theory, relaxation modulus, *E*(*t*), was generated. Then thermal stress, *σ*(*T*), and corresponding critical cracking temperature, *T_CR_*, were finally computed with three different mathematical approaches: a simple power law function, Hopkins and Hamming’s algorithm [6,37] and one-step Laplace transformation. Detailed information about theses mathematical computation approaches is provided in the next section of this study.

## 5. Mathematical Approaches for Computing Thermal Stress and Critical Cracking Temperature

### 5.1. Simple Power Law Model (Method 1)

In the case of linear viscoelastic material such as asphalt binder or mixture, creep compliance: *D*(*t*) and relaxation modulus can be intercorrelated by a simple mathematical expression as [38,39,40,41]:

(2) $$\left\{ \begin{array}{l} \int_{0}^{t}E(t-\tau )\cdot D(t)d\tau =t\\ \int_{0}^{t}E(t)\cdot D(t-\tau )d\tau =t \end{array}\right.\;$$

An approximated relationship between *D*(*t*) and *E*(*t*) exists as:

(3) E(t)⋅D(t)=1

Based on experimental experience, the approximated expression for *D*(*t*) and *E*(*t*) can be written as:

(4) $$D(t)=D_{1}\cdot t^{n},\;E(t)\;=E_{1}\cdot t^{-n}\;$$

In Equation (4), *E*_1_, *D*_1_ and *n* represent positive constants [39,41]. By taking the Laplace transformation [42,43], Equation (2) can be expressed as:

(5) $$\;L\left(\int_{0}^{t}E(t-\tau )\cdot D(t)d\tau \right)=\frac{1}{s^{2}}$$

With considerations of Gamma function, Euler reflective formula and Equations (2)–(5), the relation between *D*(*t*) and *E*(*t*) can finally be expressed as:

(6) $$E(t)\cdot D(t)=\frac{\sin(n\cdot \pi )}{n\cdot \pi }\Rightarrow E(t)=\frac{1}{D(t)}\cdot \frac{\sin(n\cdot \pi )}{n\cdot \pi }$$

In Equation (6), parameter *n* can be written as:

(7) $$n={\left|\frac{d\log D(t)}{\log\tau }\right|}_{\tau =t}\;or\;n={\left|\frac{d\log E(t)}{\log\tau }\right|}_{\tau =t}$$

Based on different research, it was found that very good *E*(*t*) prediction can be obtained when the experimental results of *D*(*t*) show a linear trend in a log–log scale (i.e., *D*(*t*) versus time) [38,41,43]. From Equations (6) and (7), *E*(*t*) master curve can be expressed as:

(8) $$E(t)=E_{g}\cdot {\left[1+{\left(\frac{t}{t_{c}}\right)}^{v}\right]}^{-\frac{w}{v}}\;$$

where:

*Eg* = glassy modulus, assumed equal to 30–40 GPa for asphalt mixtures [6,7];
*t_c_*, *ν* and *w* = fitting parameters;
*a_T_* = shift factor; this can be expressed as:
  (9) $$a_{T}=10^{C_{1}+C_{2}\cdot T_{s}}\Rightarrow \mathrm{Log}a_{T}=C_{1}+C_{2}\cdot T_{s}$$
  *C*_1_, *C_2_* = constant parameters, and
  *T_s_* = reference temperature (°C, lowPG + 10 °C).

Finally, thermal stress, *σ*(*T*), can be computed by using Equations (8) and (9) and then by solving Equation (10) numerically [6,7,12,14,16]:

(10) $$\begin{array}{l} \sigma (t)=\int_{-\infty }^{t}\dot{\varepsilon }(t)\cdot E(t-t{}^{\prime })dt{}^{\prime }\;\\ \Rightarrow \sigma (\xi )=\int_{-\infty }^{\xi }\frac{d\varepsilon (\xi {}^{\prime })}{d\xi {}^{\prime }}\cdot E(\xi -\xi {}^{\prime })d\xi {}^{\prime }=\int_{-\infty }^{t}\frac{d(\alpha \cdot \Delta T)}{dt{}^{\prime }}\cdot E(\xi (t)-\xi {}^{\prime }(t))dt{}^{\prime }\\ \Rightarrow \sigma (\xi )=\int_{-\infty }^{\xi }\frac{d\varepsilon (\xi {}^{\prime })}{d\xi {}^{\prime }}\cdot E_{g}\cdot {\left[1+{\left(\frac{\xi -\xi {}^{\prime }}{t_{c}}\right)}^{v}\right]}^{-\frac{w}{v}}d\xi {}^{\prime }=\int_{-\infty }^{t}\frac{d(\alpha \cdot \Delta T)}{dt{}^{\prime }}\cdot E_{g}\cdot {\left[1+{\left(\frac{\xi (t)-\xi {}^{\prime }(t)}{t_{c}}\right)}^{v}\right]}^{-\frac{w}{v}}dt{}^{\prime } \end{array}$$

where:

(11) $$\xi =\frac{t}{a_{T}}\Rightarrow \mathrm{Log}\xi =\mathrm{Log}t-\mathrm{Log}a_{T}=\mathrm{reduced}\;\mathrm{time}$$

*ε*(*t*) = d*ε*(*t*′)/d*t*′ = strain rate which can also be expressed as:

(12) ε(t)=α⋅ΔT

*α* = coefficient of thermal expansion or contraction assumed as *α* = 0.00003 [6,7,12,14,16], Δ*T* = temperature cooling rate (−2 °C/h and −20 °C/h were considered).

**Figure 4:**
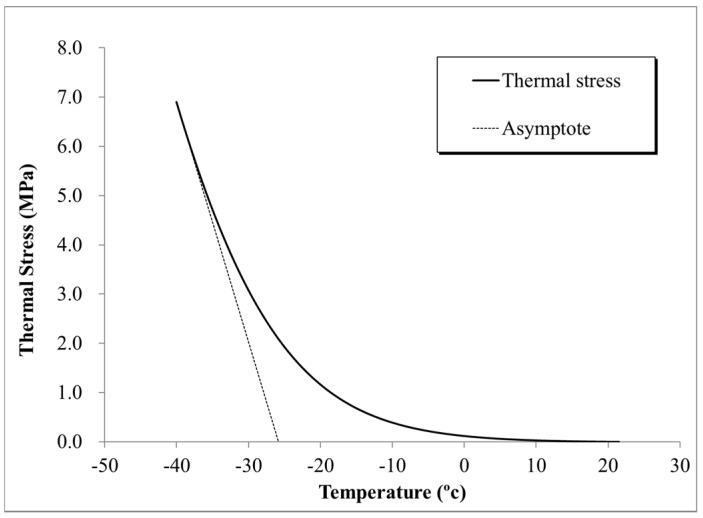
SAP (Single Asymptote Procedure) approach.

It should be mentioned that critical cracking temperature, *T_CR_*, was calculated from the results of *σ*(*T*) plot as shown in Figure 4 by using the Single Asymptote Procedure (SAP) [43]. Based, on this method, *σ*(*T*) is obtained from the intersection of the tangent to the left end of the thermal stress curve and the temperature axis.

### 5.2. Hopkins and Hamming’s Algorithm (Method 2)

In this approach, Hopkins and Hamming’s algorithm [37] was used to generate *E*(*t*) from *D*(*t*) differently from the previous section (Method 1). In this algorithm, the relationship between *E*(*t*) and *D*(*t*) can be rewritten from Equation (2) as:

(13) $$\begin{array}{l} \int_{0}^{t}E(t)\cdot D(t-\tau )d\tau =t\\ \Rightarrow \int_{t_{i}}^{t_{i}+1}E(t{}^{\prime })\cdot D(t_{n+1}-t{}^{\prime })dt{}^{\prime }=-E(t_{i+1/2})\cdot [Q(t_{n+1}-t_{i+1})-Q(t_{n+1}-t_{i})]\\ \Rightarrow E(t_{n+1/2})=\frac{t_{n+1}-\sum_{i=0}^{n-1}E(t_{i+1/2})\cdot [Q(t_{i+1})-Q(t_{i})]}{Q(t_{n+1})-Q(t_{n})} \end{array}$$

In Equation (13), *Q* can be expressed as:

(14) $$Q(t)=\int_{0}^{t}D(t)dt\Rightarrow Q(t_{n+1})=Q(t_{n})+\frac{1}{2}\cdot \left[D(t_{n+1})+D(t_{n})\right]\cdot (t_{n+1}-t_{n})$$

where *t* identifies the time interval (i.e., *t_0_* = 0, *t*_1_ = 1, *t_2_* = 2, …, *t_1000_* = 1000) which can be symbolically written as:

(15) $$t_{i+\frac{1}{2}}=\frac{1}{2}\cdot (t_{i+1}+t_{i})$$

Then, the relationship between *Q* and *E* can be expressed as:

(16) $$Q(t_{0})=0,\;E(t_{0})=0,\;E(t_{1})\;=\;\frac{t_{1}}{Q(t_{1})},\;E(t_{2})\;=\;\frac{t_{2}-E(t_{1})\cdot \left[Q(t_{2})-Q(t_{1})\right]}{Q(t_{1})}$$

After the computation of *E*(*t*) using the procedure exemplified in Equations (13) through (16), thermal stress, *σ*(*T*), can be finally computed based on Equations (8)–(11), similarly to the previous section. Moreover, the corresponding critical cracking temperature, *T_CR_*, is obtained from the SAP approach [43].

### 5.3. Laplace Transformation (Method 3)

Differently from the computation approaches mentioned in the previous section, the Laplace transformation approach provides a one-step thermal stress computation procedure without inter-converting *E*(*t*) from the experimental *D*(*t*). A brief description of the calculation process of *σ(T)* based on the Laplace transformation is presented in the following steps:

(1) From the experimental result of BBR mixture creep test [6,30,36], generate Log*D*(*t*) versus Log(*t*) curve (both at lowPG + 10 °C and low(PG + 10) − 12 °C) and compute the shift factor, *a_T_*, at a reference temperature, *T_i_* = 22 °C (see Equation (17)).
  (17) $$\left\{ \begin{array}{l} Loga_{T_{i}(\mathrm{ref}=22{}^{\circ}C)}=B_{1}+B_{2}\cdot T\\ a_{T_{i}(\mathrm{ref}=22{}^{\circ}C)}=10^{B_{1}+B_{2}\cdot T} \end{array}\right.$$
(2) Rewrite Equation (17) as follows:
  (18) $$\left\{ \begin{array}{l} a_{T}=10^{B_{1}+B_{2}\cdot T}=10^{B_{1}+B_{2}\cdot (T_{i}-B_{0}\cdot t)}=10^{(B_{1}+B_{2}\cdot T_{i})-B_{2}\cdot B_{0}\cdot t}=10^{B_{3}+B_{4}\cdot t}=10^{B_{3}}\cdot 10^{B_{4}\cdot t}=A_{0}\cdot 10^{B_{4}\cdot t}\\ B_{3}=B_{1}+B_{2}\cdot T_{i}\;,\;B_{4}=-B_{2}\cdot B_{0}\cdot t\;,A_{0}=10^{B_{3}} \end{array}\right.$$
(3) Generate master curve of *D*(*ξ*) in the reduced time domain using *D*(*t*) experimental data based on dual series of power law function as seen in Equation (19).
  (19) $$D(\xi =\frac{t}{a_{T}})=A\cdot \xi^{B}+C\cdot \xi^{D}=A\cdot {\left(\frac{t}{a_{T}}\right)}^{B}+C\cdot {\left(\frac{t}{a_{T}}\right)}^{D}$$
  where:
  *A*, *B*, *C* and *D* are fitting parameters.
  In this study, the average value of *D*(*ξ*) (i.e., average between *D*(*ξ*, at lowPG + 10 °C) and *D*(*ξ*, at lowPG + 10 − 12 °C) data were considered.
(4) Consider Equations (17)–(19) to relate thermal stress and strain assuming an idealized scheme as:
  (20) $$\varepsilon_{t}=\int_{0}^{\xi }D(\xi -\bar{\xi })\cdot \frac{\partial \sigma }{\partial \bar{\xi }}d\bar{\xi }+\int_{0}^{\xi }\alpha (\xi -\bar{\xi })\cdot \frac{\partial (\Delta T)}{\partial \bar{\xi }}d\bar{\xi }=0$$
(5) With considerations of the Laplace transformation, Equation (20) can be expressed as:
  (21) $$\begin{array}{l} \;L\left(\varepsilon_{t}\right)=L\left(\int_{0}^{\xi }D(\xi -\bar{\xi })\cdot \frac{\partial \sigma }{\partial \bar{\xi }}d\bar{\xi }+\int_{0}^{\xi }\alpha (\xi -\bar{\xi })\cdot \frac{\partial (\Delta T)}{\partial \bar{\xi }}d\bar{\xi }\right)\\ =s\cdot \bar{D}(s)\cdot \bar{\sigma }(s)+s\cdot \bar{\alpha }(s)\cdot \Delta \bar{T}(s)=0\\ \to \bar{\sigma }(s)=-\frac{\bar{\alpha }(s)\cdot \Delta \bar{T}(s)}{\bar{D}(s)} \end{array}$$
  Based on Equations (17)–(21) parameter *α* and *T* can be reexpressed as:
  (22) $$\left\{ \begin{array}{l} \alpha (t)=\alpha_{0}\Rightarrow L\alpha (t)=\bar{\alpha }(s)=\frac{\alpha_{0}}{s}\\ \Delta T(t)=-B_{0}\cdot t\Rightarrow L\Delta T(t)=\Delta \bar{T}(s)=-\frac{B_{0}}{s^{2}} \end{array}\right.\;$$
(6) Perform inverse Laplace transformation of Equations (21) and (22) using the Stehfest algorithm [37,39,40,44,45]. Then, fit the results of *σ*(*ξ*) using a simple power law function as:
  (23) $$\sigma (\xi )=A+B\cdot \xi^{C}$$
(7) Compute thermal stress, (T) in the actual time domain, starting from the thermal stress: *σ*(*ξ*) in the reduced time domain using Equations (17)–(24).
  (24) $$\left\{ \begin{array}{l} \xi =\int_{0}^{t}\frac{dt{}^{\prime }}{a_{T}[T(t{}^{\prime })]}=\int_{0}^{t}\frac{dt{}^{\prime }}{A_{0}\cdot 10^{B_{4}\cdot t{}^{\prime }}}=\frac{1}{A_{0}\cdot B_{4}\cdot \ln10}\cdot \left[1-10^{-B_{4}t}\right]=A_{1}\cdot \left[1-10^{-B_{4}t}\right]\\ A_{1}=\frac{1}{A_{0}\cdot B_{4}\cdot \ln10} \end{array}\right.$$

By using Equations (17)–(24), the time–domain thermal stress, *σ*(*T*) can be finally calculated between 22 and −40 °C with a temperature step of 0.5 °C. It also needs to be mentioned that corresponding critical cracking temperature, *T_CR_*, can be obtained, as for the approaches adopted in Section 5.1 and Section 5.2, with the SAP method [43].

## 6. Data Analysis

### 6.1. Creep Stiffness and m-Value

**Figure 5:**
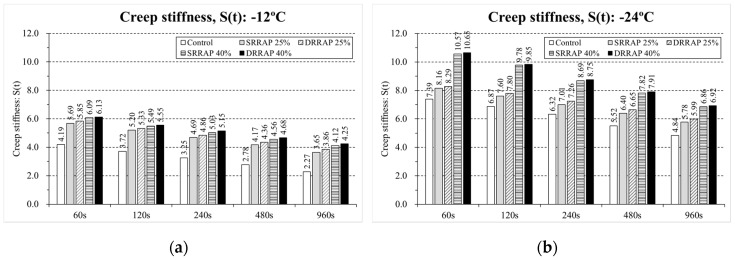
Creep stiffness, *S*(*t*), at different testing time. (**a**) T = −12 °C; (**b**) T = −24 °C.

**Figure 6:**
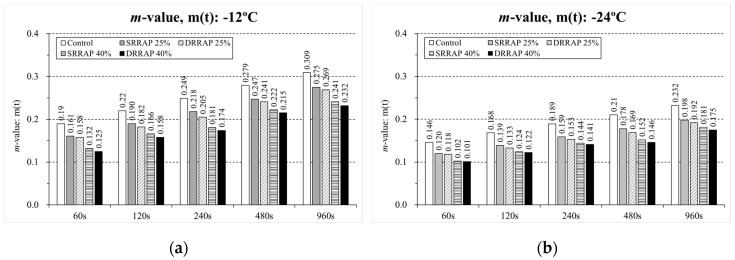
*m*-value, *m*(*t*), at different testing time. (**a**) T = −12 °C; (**b**) T = −24 °C.

Based on Equation (1), creep stiffness, *S*(*t*) and *m*-value, *m*(*t*), were computed and compared at different testing times as reported in the bar charts presented in Figure 5 and Figure 6.

As could be expected, the addition of RAP resulted in a higher creep stiffness and lower *m*-values due to the increasing degree of brittleness introduced by the recycled material. In the case of *S*(*t*), higher differences were observed for each testing time at the lower testing temperature (−24 °C) when comparing the material designed with a RAP content of 25% and 40%. However, an opposite trend was exhibited from *m*(*t*) with more remarkable differences at the higher temperature (−12 °C). Form a visual comparison, it also appears that, for the two distinct degrees of recycling (25% and 40%), a relatively moderate difference can be detected between SRRAP and DRRAP mixtures at both testing temperatures. It can be noted that the values of the coefficient of variation (also known as relative standard deviation) of the BBR measurements are in most cases below 10% and overall lower than a commonly accepted threshold of 15% for BBR tests on mixtures (Table 3 and Table 4). This confirms the repeatability of this experimental method as already demonstrated in previous research [14,16,30,31]. To further evaluate the response of the material at low temperature, the results of the thermal stress calculation and critical cracking temperature are reported in the next sections of this paper.

### 6.2. Thermal Stress

In this part, of the present manuscript, the results of the thermal stress computation obtained from the three different models are presented and graphically compared. Then, a new thermal stress index is introduced to provide a simple tool for performing a quantitative comparison between the different materials concerning their low-temperature behavior.

#### 6.2.1. Thermal Stress Curves

**Figure 7:**
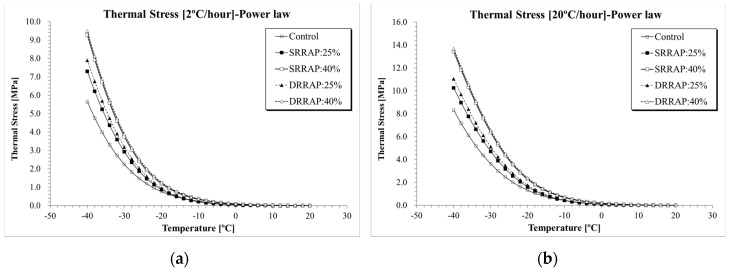
*σ*(*T*) computation results (Simple Power law): (**a**) 2 °C/h; (**b**) 20 °C/h.

**Figure 8:**
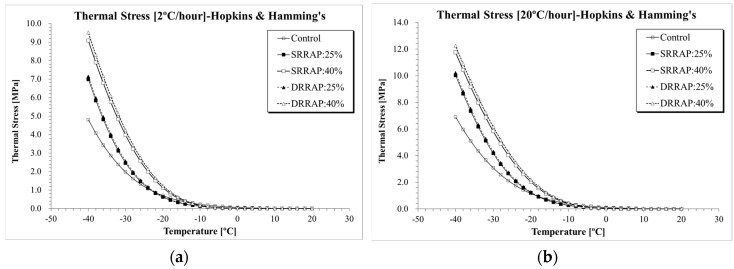
*σ*(*T*) computation results (Hopkins and Hamming, 1957): (**a**) 2 °C/h; (**b**) 20 °C/h.

**Figure 9:**
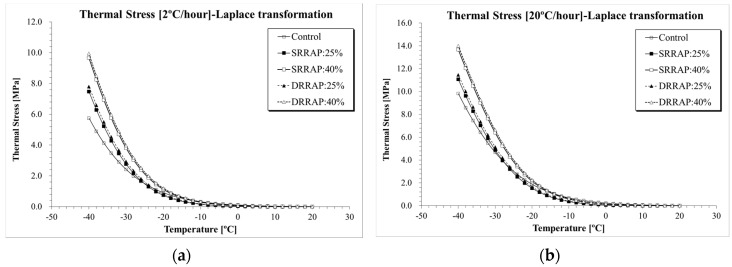
*σ*(*T*) computation results (Laplace transformation): (**a**) 2 °C/h; (**b**) 20 °C/h.

Thermal stress was computed for each asphalt mixture under two distinct cooling rates (e.g., −2 and −20 °C/h) using Equations (1)–(24). Figure 7, Figure 8 and Figure 9 present the thermal stress curves of control, SRRAP and DRRAP mixtures.

Based on the plots, in all cases, mixtures containing 25% or 40% of RAP presented higher thermal stress than the virgin material. Approximately 30–45% increase in *E*(*t*) was observed for mixtures containing 25% of RAP when compared to the control mixture. A higher increment in thermal stress between 40% and 97% was experienced when 40% of RAP was incorporated in the mix design. While this indicates potentially poorer performance at low temperatures for asphalt mixtures containing recycled materials, only moderate differences in the rage of 2–7% were observed between SRRAP and DRRAP mixtures at both recycling levels (25% and 40% of RAP). Such a limited difference seems to suggest that comparable performance may be expected at low temperatures from single and double-recycled mixtures when considering the specific mix design, RAP material and content used in the present experimental effort. The potentially detrimental effect of RAP on low-temperature response was observed in several previous studies [6,7,12,16]. However, based on the experimental results and assuming the level of performance of SRRAP mixtures acceptable, it appears that mixture of second recycling generation, DRRAP, may deserve further research consideration for future possible application in wearing courses depending on traffic volume, allocated budget, construction costs and expected pavement service life.

**Figure 10:**
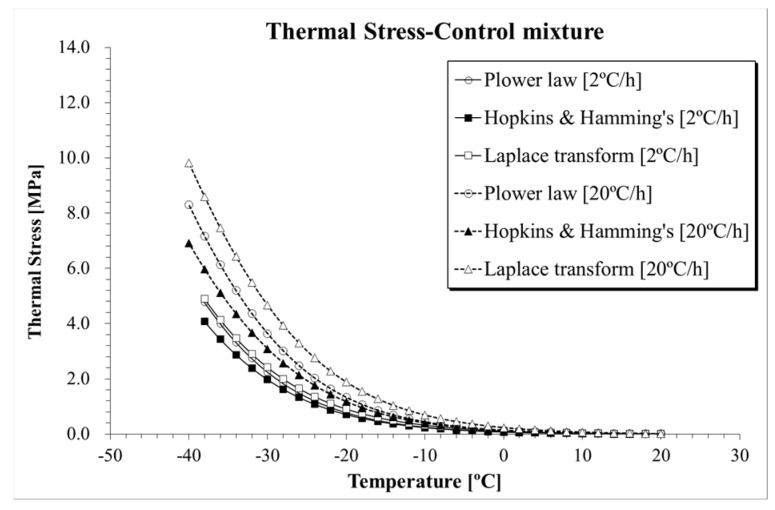
*σ*(*T*) computation results (Control mixture).

**Figure 11:**
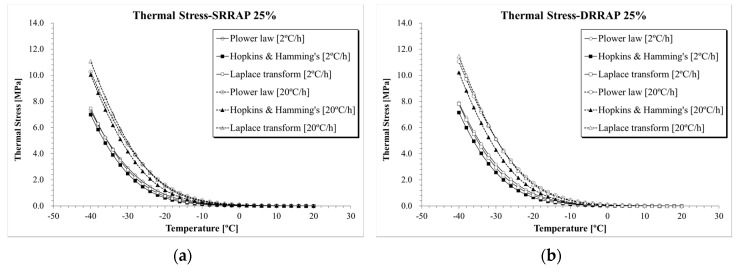
*σ*(*T*) computation results: (**a**) SRRAP 25% mixture; (**b**) DRRAP 25% mixture.

**Figure 12:**
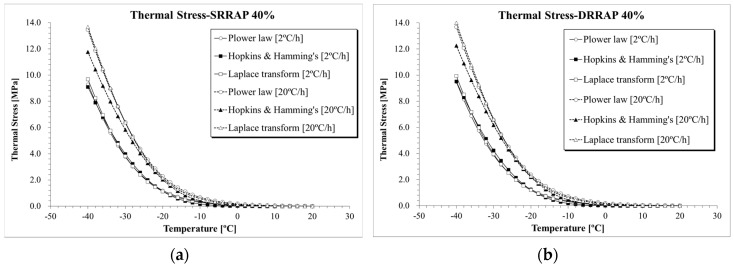
*σ*(*T*) computation results: (**a**) SRRAP 40% mixture; (**b**) DRRAP 40% mixture.

The results of thermal stress for the five asphalt mixtures obtained from the three selected computation approaches are directly compared in Figure 10, Figure 11 and Figure 12. Clear differences in thermal stresses among the different computation solutions can be observed. In all cases, the highest, intermediate and lowest thermal stresses were found when Laplace transformation, power law function and Hopkins and Hamming’s algorithm [37] approach were applied, respectively. From a practical point of view, given the relatively close level of predictions of the three formulations, the three mathematical approaches could be used to provide upper and lower bounds of the thermal stress prediction.

In the next section, a thermal stress factor is proposed for performing a simpler and easy comparison of the response of the mixture at low temperatures.

#### 6.2.2. Thermal Stress Factor (TSF)

**Figure 13:**
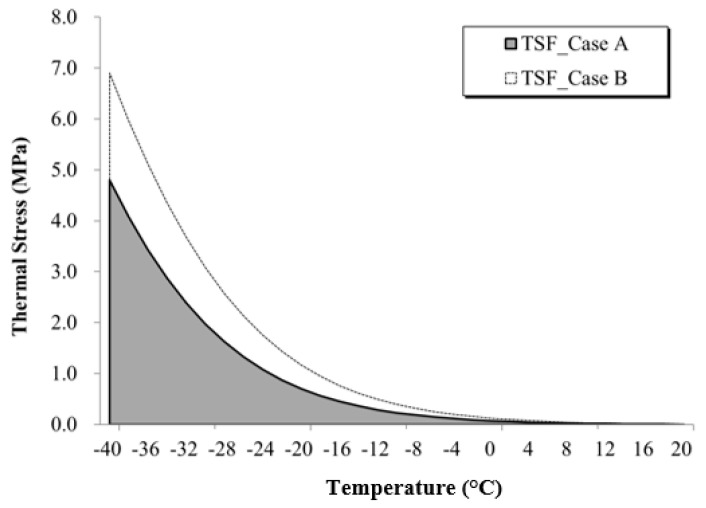
Schematic of the thermal stress factor (*TSF*) computation.

For bituminous material such asphalt mixture, amount and rate of increase (i.e., slope) of thermal stress are crucial. It is well known that a steeper increase rate of thermal stress provides negative performance on asphalt mixture at low temperatures. In this paper, a new thermal stress indicator for asphalt mixture named thermal stress factor (*TSF*) is introduced. This parameter is defined as the ratio between the difference in the areas under the thermal stress curve for the material of interest and the reference material in percentage (see Figure 13):

(25) $$TSF(\%)=\frac{A_{M}-A_{R}}{A_{R}}\times 100=\frac{\int_{T_{i}}^{T_{f}}\sigma_{M}(T)dT-\int_{T_{i}}^{T_{f}}\sigma_{R}(T)dT}{\int_{T_{i}}^{T_{f}}\sigma_{R}(T)dT}\times 100$$

where *A_M_* and *A_R_* are the areas under the thermal stress curve for the material of interest and the reference material; *σ_M_*(*T*) and *σ_R_*(*T*) are the thermal stress for the material of interest and the reference material; *T_i_* and *T_f_* are the initial and final integration temperatures corresponding to *T_i_* = 20 °C and *T_f_* = −40 °C in the present paper as defined when computing the thermal stress.

The TSF is based on the computation of the area under the thermal stress curve of the specific mixture. A smaller area corresponds to relatively better performance against thermal cracking. The values of *TSF* are shown in Table 5 for the entire set of mixtures and thermal stress calculation methods. It must be noted that in the case of the *TFS* computed for mixtures C and E (DRRAP), the reference material was set to the corresponding SRRAP mixture for the respective RAP content (25% and 40%). This was done to obtain a direct comparison between the first and second levels of recycling. In a different work, a similar concept was adopted for defining a binder rheological index associated with aging; in that research effort, the area between master curves of shear modulus as function frequency was used [46].

The results reported in Table 3 indicate a remarkable increase in *TSF* when RAP is incorporated in the mix design from the first round of recycling. On the other hand, a limited difference in *TFS* can be observed between SRRAP and DRRAP mixtures supporting the considerations derived in the previous section on further investigation of the double recycling process when material performance is met. Moreover, the distinct *TSF*s obtained from the three different thermal stress computation approaches (e.g., power law, Hopkins and Hamming’s and Laplace transformation) appear to confirm that these three formulations could be used to provide upper and lower bounds on low-temperature performance prediction of given asphalt mixtures.

### 6.3. Comparisons on Critical Cracking Temperature

Along with thermal stress, critical cracking temperature, *T_CR_*, was also computed based on the SAP method [37] and then compared as shown in Table 6 and Table 7.

The highest *T_CR_* was found for the control mixture and lowest *T_CR_* was obtained in the case of DRRAP 40% mixture. Moreover, lower *T_CR_* was computed for DRRAP mixture (e.g., 25% and 40%). In the previous section, the highest *σ*(*T*) was found when the Laplace transformation was applied. On the other hand, no consistent ranking of *T_CR_* could be derived for the different computation approaches of the thermal stress curves. It needs to be remarked that *T_CR_* was derived using the SAP method [43]; this provides an estimate of *T_CR_* when, as in this case, no low-temperature strength tests can be performed.

## 7. Summary and Conclusions

In this study, the effect of a second cycle of recycling on the low-temperature performance of asphalt mixtures was experimentally investigated and analyzed based on three different mathematical formulations for the computation of thermal stress. Single and double-recycled RAP (SRRAP and DRRAP) were first artificially produced in the laboratory and incorporated in the recycled mixtures at two different levels: 25% and 40%. Virgin, SRRAP and DRRAP mixtures were then subjected to low-temperature creep tests and the results analyzed using a simple power law function, the Hopkins and Hamming’s algorithm and the Laplace transformation. In the end, a simple low-temperature indicator named thermal stress factor (*TFS*) was introduced and used for further analysis. Based on the adopted research approach the following conclusions can be drawn:

- BBR mixture creep results indicate that mixtures prepared with SRRAP have almost identical low-temperature cracking performance to that of mixtures designed with DRRAP. As expected, all RAP mixtures (SRRAP or DRRAP) presented a poorer performance at low temperatures compared to the conventional asphalt mixtures;
- By applying different mathematical approaches, upper and lower bounds on thermal stress can be derived. In the case of critical cracking temperature, the different mathematical approaches did not provide clear upper and lower bounds. In this sense, the use of strength or fracture tests is recommended to verify if the three different formulations may result in a consistent critical cracking temperature;
- The *TSF* values support the trends obtained from the experimentation and provide a simple tool that could be potentially used on a routine basis from practitioners to compare the response of mixtures against low-temperature cracking. In this view, the three mathematical models provide the link between the experimental measurements and the practical information obtained from the TSF.

Due to the stiffer and more brittle characteristics, recycled mixtures with a high amount of RAP (SRRAP or DRRAP) are not recommended for surface layers in the current asphalt pavement highway projects in South Korea. However, in the present study, the possibility of using RAP (SRRAP or DRRAP) mixtures for surface or binder layers was investigated in specific research conditions with promising results. This supports the idea of evaluating DRRAP mixtures in the field through experimental pavement sections. Nevertheless, it should be mentioned that only limited asphalt mixtures with re-recycled RAP were prepared and tested in this research. Additional materials and experimental work, pavement structure analysis, including finite element method (FEM) simulation, microstructural investigation combined with mathematical and statistical evaluation need to be performed to further evaluate the effect of re-recycled RAP for pavement mixtures. This is part of a current research effort that aims to provide simple, cost-efficient and environmentally friendly recycling solutions to the pavement industry. The next step of this research will address different mixture types with a wider range of recycling combinations.

## Figures and Tables

**Table 1:** Asphalt mixtures.

Mix ID	Recycling Level	Asphalt Binder (PG)	RAP (%)	RAP Origin Mixture	NMAS (mm)	Aggregate Gradation Size (mm)/Passing (%)	Target Air Voids (%)
A	Reference (virgin)	64–22	0	–	12.5	(13/91), (10/82), (5/60), (2.5/41), (0.6/22), (0.3/16), (0.15/10), (0.08/6)	4.2–4.9
B	SRRAP ^1^	64–22	25	A	12.5	(13/92), (10/78), (5/55), (2.5/35), (0.6/20), (0.3/15), (0.15/11), (0.08/5)	4.2–4.9
C	SRRAP	64–22	40	A	12.5	(13/91), (10/77), (5/54), (2.5/37), (0.6/22), (0.3/17), (0.15/12), (0.08/6)	4.2–4.9
D	DRRAP ^2^	64–22	25	B	12.5	(13/92), (10/79), (5/53), (2.5/35), (0.6/23), (0.3/16), (0.15/11), (0.08/5)	4.2–4.9
E	DRRAP	64–22	40	C	12.5	(13/91), (10/81), (5/50), (2.5/34), (0.6/24), (0.3/15), (0.15/11), (0.08/5)	4.2–4.9

^1^ single recycled reclaimed asphalt pavement, ^2^ double-recycled reclaimed asphalt pavement.

**Table 2:** Schematic information for BBR mixture testing.

Test	Mix ID	Asphalt Binder PG	Testing Temperature (°C) (Number of Replicates)	Applied Load (mN) (Testing Temperature °C)
BBR	A, B, C, D, E	PG 64–22	low(PG + 10 °C) = −12 °C (10) low(PG + 10 °C) – 12 °C = −24 °C (10)	4000-mN (−12 °C) 6000-mN (−24 °C)

**Table 3:** Results of *S*(*t*) at *t* = 60, 120, 240, 480, 960 s.

Mix ID	RAP (%)	Creep Stiffness: *S*(*t*) (GPa), Coefficient of Variation, (%)									
		60 s		120 s		240 s		480 s		960 s	
		−12 °C	−24 °C	−12 °C	−24 °C	−12 °C	−24 °C	−12 °C	−24 °C	−12 °C	−24 °C
A	Control (0%)	4.19 (9.4%)	7.39 (10.2%)	3.72 (8.8%)	6.87 (8.2%)	3.25 (9.6%)	6.32 (9.5%)	2.78 (9.4%)	5.52 (10.1%)	2.27 (7.6%)	4.84 (7.2%)
B	SRRAP (25%)	5.69 (8.2%)	8.16 (9.5%)	5.20 (9.8%)	7.60 (10.1%)	4.69 (8.7%)	7.07 (8.8%)	4.17 (10.5%)	6.40 (9.2%)	3.65 (9.1%)	5.78 (8.1%)
C	SRRAP (40%)	6.09 (9.9%)	10.57 (8.5%)	5.49 (8.1%)	9.78 (9.5%)	5.03 (7.4%)	8.69 (9.3%)	4.56 (9.6%)	7.82 (9.8%)	4.12 (9.2%)	6.86 (7.9%)
D	DRRAP (25%)	5.85 (8.5%)	8.29 (9.2%)	5.33 (8.4%)	7.80 (9.5%)	4.86 (9.1%)	7.26 (8.8%)	4.36 (7.8%)	6.65 (8.4%)	3.86 (8.3%)	5.99 (8.7%)
E	DRRAP (40%)	6.13 (9.5%)	10.65 (8.8%)	5.55 (9.3%)	9.85 (9.2%)	5.15 (8.7%)	8.75 (9.4%)	4.68 (8.4%)	7.91 (9.9%)	4.25 (8.6%)	6.92 (8.2%)

( )—coefficient of variation; SRRAP—single recycled reclaimed asphalt pavement; DRRAP—double-recycled reclaimed asphalt pavement.

**Table 4:** Results of *m*(t) at *t* = 60, 120, 240, 480, 960 s.

Mix ID	RAP (%)	*m*-Value: *m*(*t*) = d*S*(*t*)/d*t*, Coefficient of Variation (%)									
		60 s		120 s		240 s		480 s		960 s	
		−12 °C	−24 °C	−12 °C	−24 °C	−12 °C	−24 °C	−12 °C	−24 °C	−12 °C	−24 °C
A	Control (0%)	0.190 (9.7%)	0.146 (10.6%)	0.220 (8.5%)	0.168 (8.7%)	0.249 (9.7%)	0.189 (8.5%)	0.279 (10.0%)	0.210 (9.5%)	0.309 (7.9%)	0.232 (8.6%)
B	SRRAP (25%)	0.161 (9.9%)	0.120 (9.8%)	0.190 (9.3%)	0.139 (9.1%)	0.218 (9.4%)	0.159 (8.8%)	0.247 (10.5%)	0.178 (9.1%)	0.275 (7.7%)	0.198 (9.5%)
C	SRRAP (40%)	0.132 (9.1%)	0.102 (8.1%)	0.166 (9.9%)	0.124 (7.5%)	0.181 (8.4%)	0.144 (9.2%)	0.222 (10.1%)	0.152 (9.5%)	0.241 (8.4%)	0.181 (9.2%)
D	DRRAP (25%)	0.158 (8.4%)	0.118 (8.6%)	0.182 (9.4%)	0.133 (8.4%)	0.205 (8.1%)	0.153 (8.4%)	0.241 (7.1%)	0.169 (9.5%)	0.269 (7.4%)	0.192 (8.5%)
E	DRRAP (40%)	0.125 (8.8%)	0.101 (8.3%)	0.158 (9.8%)	0.122 (8.9%)	0.174 (9.5%)	0.141 (8.8%)	0.215 (8.4%)	0.146 (9.9%)	0.232 (8.6%)	0.175 (9.2%)

( )—coefficient of variation; SRRAP—single recycled reclaimed asphalt pavement; DRRAP—double-recycled reclaimed asphalt pavement.

**Table 5:** Thermal stress factor (TSF) computation results.

ID	Mixture	Approach	Area (MPa·°C)		*TFS* (%) [Mix_Reference_] − [Mix_Interest_]	
			2 °C/h	20 °C/h	2 °C/h	20 °C/h
A	Control	PL	58.0	92.5	– –	– –
		HH	50.9	78.6	– –	– –
		LA	62.9	115.4	– –	– –
B	SRRAP 25%	PL	72.7	114.8	25.3 PL: [B] − [A]	24.1 PL: [B] − [A]
		HH	62.5	101.6	22.8 HH: [B] − [A]	29.3 HH: [B] − [A]
		LA	69.5	119.4	10.5 LA: [B] − [A]	3.5 LA: [B] − [A]
D	DRRAP 25%	PL	78.8	124.3	8.4 PL: [C] − [B]	8.3 PL: [C] − [B]
		HH	64.7	104.6	3.5 HH: [C] − [B]	3.0 HH: [C] − [B]
		LA	73.9	124.6	6.3 LA: [3] − [2]	4.4 LA: [3] − [2]
C	SRRAP 40%	PL	95.0	156.8	63.8 PL: [D] − [A]	69.5 PL: [D] − [A]
		HH	93.3	136.4	83.3 HH: [D] − [A]	73.5 HH: [D] − [A]
		LA	95.6	154.9	52.0 LA: [D] − [A]	34.2 LA: [D] − [A]
E	DRRAP 40%	PL	98.4	161.2	3.6 PL: [E] − [D]	2.8 PL: [E] − [D]
		HH	99.2	144.2	6.3 HH: [E] − [D]	5.7 HH: [E] − [D]
		LA	99.7	160.5	4.3 LA: [E] − [D]	3.6 LA: [E] − [D]

PL—simple power law; HH—Hopkins and Hamming; LA—Laplace transformation.

**Table 6:** Results of *T_CR_* (°C) based on the SAP approach (2 °C/hour).

Mixture	*T_CR_* (°C)			*T_CR_* Ranking
	PL	HH	LA	
Control	−27.7	−27.3	−27.2	LA > HH > PL
SRRAP 25%	−26.9	−28.2	−27.7	PL > LA > HH
SRRAP 40%	−26.6	−25.3	−26.7	HH > PL > LA
DRRAP 25%	−26.7	−28.1	−27.5	PL > LA > HH
DRRAP 40%	−26.5	−25.0	−26.5	HH > PL = LA

PL—power law; HH—Hopkins and Hamming’s algorithm; LA—Laplace transformation.

**Table 7:** Results of *T_CR_* (°C) based on the SAP approach (20 °C/hour).

Mixture	*T_CR_* (°C)			*T_CR_* Ranking
	PL	HH	LA	
Control	−25.9	−25.8	−25.9	HH > LA = PL
SRRAP 25%	−24.5	−25.8	−24.5	PL = LA > HH
SRRAP 40%	−23.3	−22.8	−23.3	HH > PL = LA
DRRAP 25%	−24.2	−25.7	−24.2	PL = LA > HH
DRRAP 40%	−23.1	−22.5	−23.1	HH > PL = LA

PL—power law; HH—Hopkins and Hamming’s algorithm; LA—Laplace transformation.
